# Harm reduction and viral hepatitis C in European prisons: a cross-sectional survey of 25 countries

**DOI:** 10.1186/s12954-018-0230-1

**Published:** 2018-05-11

**Authors:** Rob Bielen, Samya R. Stumo, Rachel Halford, Klára Werling, Tatjana Reic, Heino Stöver, Geert Robaeys, Jeffrey V. Lazarus

**Affiliations:** 1 Faculty of Medicine and Life Sciences, Hasselt University, Hasselt, Belgium; 2 Department of Gastroenterology and Hepatology, Ziekenhuis Oost Limburg, Genk, Belgium; 3 Barcelona Institute for Global Health (ISGlobal), Hospital Clínic, University of Barcelona, Carrer del Roselló, 132, 4th, ES-08036 Barcelona, Spain; 4 The Hepatitis C Trust, London, UK; 5 2nd Department of Internal Medicine, Semmelweis University, Budapest, Hungary; 6 European Liver Patients’ Association (ELPA), Brussels, Belgium; 7 Institute for Addiction Research, Frankfurt University of Applied Sciences, Frankfurt, Germany; 8 Department of Gastroenterology and Hepatology, University Hospitals KU Leuven, Leuven, Belgium; 9 CHIP, Rigshospitalet, University of Copenhagen, Copenhagen, Denmark

**Keywords:** Cross-sectional survey, Harm reduction, Hepatitis C, Injecting drug use, Needle and syringe program, Opioid substitution therapy, Policy monitoring, Prison health, Europe

## Abstract

**Background:**

Current estimates suggest that 15% of all prisoners worldwide are chronically infected with the hepatitis C virus (HCV), and this number is even higher in regions with high rates of injecting drug use. Although harm reduction services such as opioid substitution therapy (OST) and needle and syringe programs (NSPs) are effective in preventing the further spread of HCV and HIV, the extent to which these are available in prisons varies significantly across countries.

**Methods:**

The Hep-CORE study surveyed liver patient groups from 25 European countries in 2016 and mid-2017 on national policies related to harm reduction, testing/screening, and treatment for HCV in prison settings. Results from the cross-sectional survey were compared to the data from available reports and the peer-reviewed literature to determine the overall degree to which European countries implement evidence-based HCV recommendations in prison settings.

**Results:**

Patient groups in nine countries (36%) identified prisoners as a high-risk population target for HCV testing/screening. Twenty-one countries (84%) provide HCV treatment in prisons. However, the extent of coverage of these treatment programs varies widely. Two countries (8%) have NSPs officially available in prisons in all parts of the country. Eleven countries (44%) provide OST in prisons in all parts of the country without additional requirements.

**Conclusion:**

Despite the existence of evidence-based recommendations, infectious disease prevention measures such as harm reduction programs are inadequate in European prison settings. Harm reduction, HCV testing/screening, and treatment should be scaled up in prison settings in order to progress towards eliminating HCV as a public health threat.

## Background

At any given moment, an estimated 1.6 million men, women, and children are in prison throughout the 53 Member States of the World Health Organization (WHO) European Region [1]. Furthermore, due to high turnover in prison populations, an estimated 6 million people in total are incarcerated at some point during a given year in these 53 countries [2]. The prevalence of hepatitis C virus (HCV) infection among prisoners is many times higher than in the general population. The HCV prevalence in the general population in Europe ranges from 0.5% in Western Europe to 2.5% in Southern Europe and reaches 6% in Eastern Europe [3]. In prisons, the estimated prevalence is 15.4% in Western Europe and 20.7% in Eastern Europe [4]. However, these estimates are based on few studies and could even be an underestimation, as shown in studies from Ukraine [5, 6].

There is a strong association between imprisonment, injecting drug use, and HCV infection [7, 8]. The mean incidence of HCV infection among prisoners with a history of injecting drug use is 16.4 cases per 100 person-years [9]. This is disproportionately high in Europe as well as in other parts of the world. In Australian prisons, between 33.3 and 23.2% (rates in the study fell from 2004 to 2010 as injecting drug use declined nationwide) of prisoners entering incarceration were found to be HCV antibody positive (HCV Ab+). This increased to 57.2% among entering inmates who reported injecting drugs. Of the inmates who were HCV Ab+, 33.7% had been unaware of their infection at the time of screening [10].

Harm reduction services such as needle and syringe programs (NSPs) and opioid substitution therapy (OST) have been identified as important interventions to combat the high rates of HCV and HIV in people who inject drugs (PWID) [11, 12]. Furthermore, providing safe alternatives for other prison-related harmful practices such as sharing of tattooing and body-piercing equipment, sharing of razors, and blood-sharing or “brotherhood” rituals has additional potential to reduce the transmission of blood-borne infection [13, 14]. Harm reduction practices have been widely considered to be a practical, effective, and economical way to reduce health-related harm caused by injecting drug use [11, 15–17]. Improved public health and a commitment to human rights are frequently cited as the primary reasons for implementing harm reduction services for PWID [18, 19]. According to the principles of universal human rights, HCV prevention, testing, treatment, and care should be widely accessible not only outside of prisons but also to the prison population [18, 20–23].

Therefore, in 2014, Arain et al. developed specific recommendations for the management of HCV in prisons to complement current recommendations for viral hepatitis based on a comprehensive review of the available literature. These recommendations include addressing each of the aforementioned issues in addition to the provision of specific harm reduction programs, scaled-up health education activities, and a multidisciplinary response [24]. Ranieri et al. later updated these recommendations and specifically addressed the issue of direct-acting antiviral (DAA) therapy in prison [25]. Recently, the Hepatitis B and C Public Policy Association published policy guidelines for HCV elimination in Europe. These guidelines notably referred to prisoners as a key focus population for establishing integrated care pathways and overcoming specific health system barriers related to the management and eventual elimination of hepatitis C infection in Europe [26].

To date, there are scarce data on the degree of implementation of key, evidence-based HCV recommendations in prisons, either in Europe or globally [18, 27–29]. This study is the first pan-European study on the availability of hepatitis care in penitentiary settings, and it specifically focuses on harm reduction, HCV testing/screening, and treatment in prisons.

## Methods

The Hep-CORE study was commissioned by the European Liver Patients’ Association (ELPA) in 2015 to determine the extent to which European countries were adhering to international policy guidelines for viral hepatitis [30]. The original cross-sectional study was conducted in 2016 and employed a questionnaire with 39 closed-ended questions across seven focal topics in viral hepatitis policy [31].

In mid-2017, a follow-up study was conducted. Hep-CORE 2017 was designed to provide a benchmark against which to measure future changes in each of the 25 European countries involved in the study and consisted of 11 main questions which were a subset of the original 2016 questionnaire. The study instruments for the project were created and managed using the web-based online data collection tool Research Electronic Data Capture (REDCap) [32]. Sampling was purposive as the respondent cohort was limited to patient groups from each of the 25 European countries with active ELPA member organizations (Table 1).

**Table 1:** Twenty-five European countries with patient group respondents in the Hep-CORE Study

Austria	Greece	Slovakia
Belgium	Hungary	Slovenia
Bosnia and Herzegovina	Italy	Spain
Bulgaria	Macedonia	Sweden
Croatia	Netherlands	Turkey
Denmark	Poland	Ukraine
Finland	Portugal	UK
France	Romania	
Germany	Serbia	

For this study, we selected data from three questions that refer to harm reduction, testing/screening, and treatment in penitentiary settings. We used the most recent data available from the Hep-CORE study. Data on harm reduction and HCV treatment in prisons are from the 2017 results, while data on HCV testing in prisons are from the 2016 dataset as that question was not repeated in the 2017 follow-up study. Patient group responses to the questionnaire were cross-referenced with the published literature from PubMed and EMBASE by searching with the following keywords: hepatitis C, prison, testing, screening, treatment, harm reduction, opioid substitution therapy, methadone, suboxone, and needle and syringe program. Search results were systematically screened by two authors. The last literature search was conducted in January 2018. These data were again compared to the *Global state of harm reduction 2016* [18].

The literature was graded in order to quantify the reliability of the evidence and implementation in the country (Table 2). Grading was impacted by whether data collection occurred before or after the start of 2015, the year following the publication of the recommendations for the management of HCV in prisons [24]. Where discrepancies between Hep-CORE data and the literature were found, results were again checked by follow-up with the participating patient groups. When patient groups did not respond to follow-up, we sought out experts where available to supplement the gaps in knowledge. Where the literature could not confirm patient group responses, the level of agreement was coded as “some disagreement” because the sources could not be uniformly verified against one another, and the majority response was coded as the result.

**Table 2:** Grading for the peer-reviewed literature

Grade A	Multicenter trial Data collection after 2015
Grade B	Monocenter trial Data collection after 2015
Grade C	Multicenter trial Data collection before 2015
Grade D	Monocenter trial Data collection before 2015

## Results

### Hepatitis C testing/screening

**Fig. 1:**
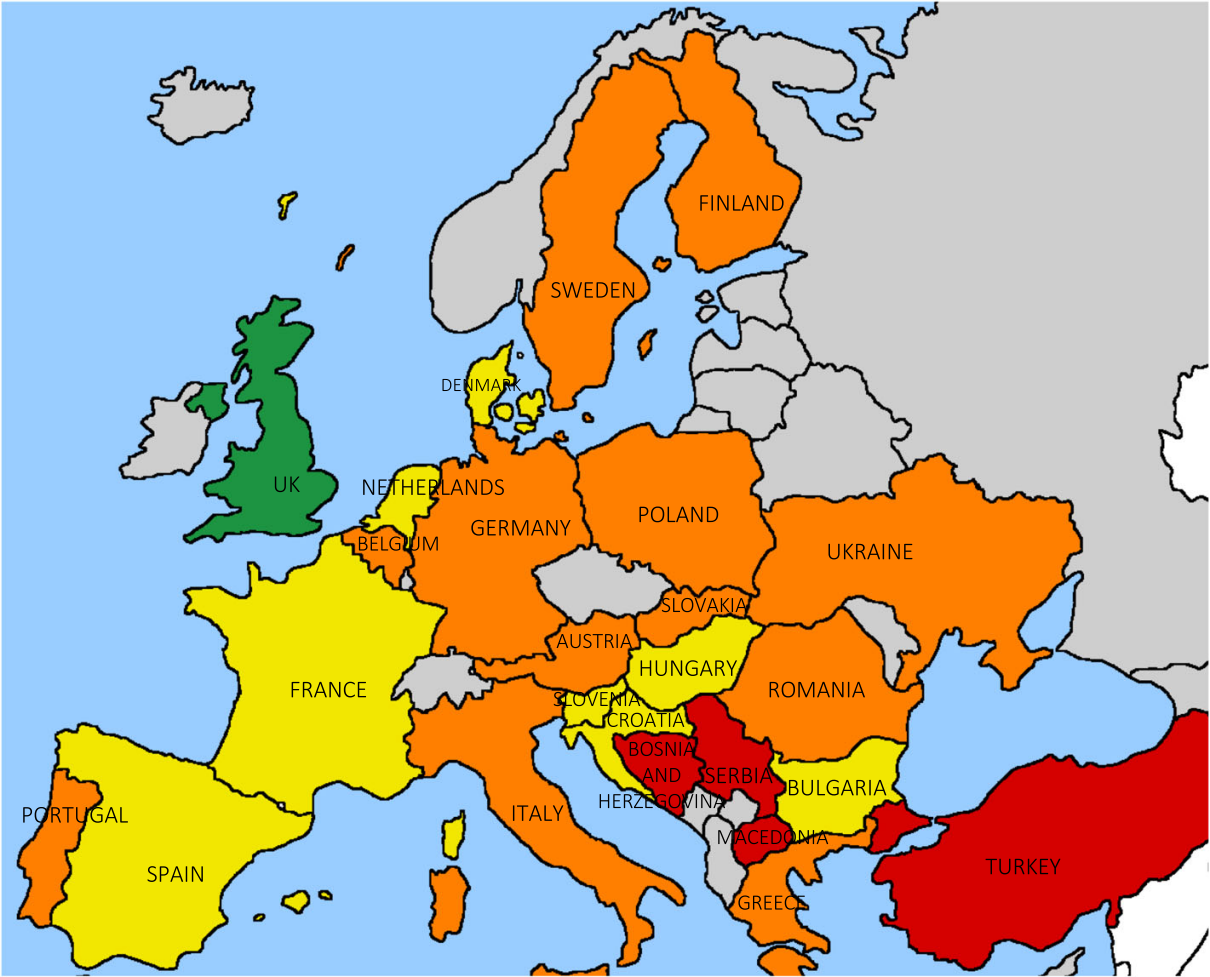
Availability and coverage of HCV testing/screening in European prisons. *Green*: universal screening in prison upon entry, opt-out procedure; *yellow*: testing/screening for HCV available in prison, extent unknown, but highlighted as an at-risk population for HCV; *orange*: testing/screening available in prison, extent unknown, not highlighted as an at-risk population for HCV; *red*: no data available from the literature, prisoners not highlighted as an at-risk population for HCV; *gray*: not part of the Hep-CORE dataset

Testing/screening for HCV is provided in at least one prison in 21 (84%) of the 25 countries included in this study (Fig. 1). However, in 16 countries (64%), patient groups reported that there was no specific HCV screening policy for prisoners as a high-risk population. Though testing might be available in prison settings, it was reported to be offered only if requested by the prisoner or if a medical doctor proposed it. Therefore, coverage of testing is considered low overall.

In nine countries (36%; Bulgaria, Croatia, Denmark, France, Hungary, the Netherlands, Slovenia, Spain, United Kingdom [UK]), prisoners were identified as a high-risk population target for HCV testing/screening. In the UK specifically, as of 2016, a universal opt-out screening procedure upon prison admission has been implemented in prisons and coverage is being monitored [33].

### Hepatitis C treatment

**Fig. 2:**
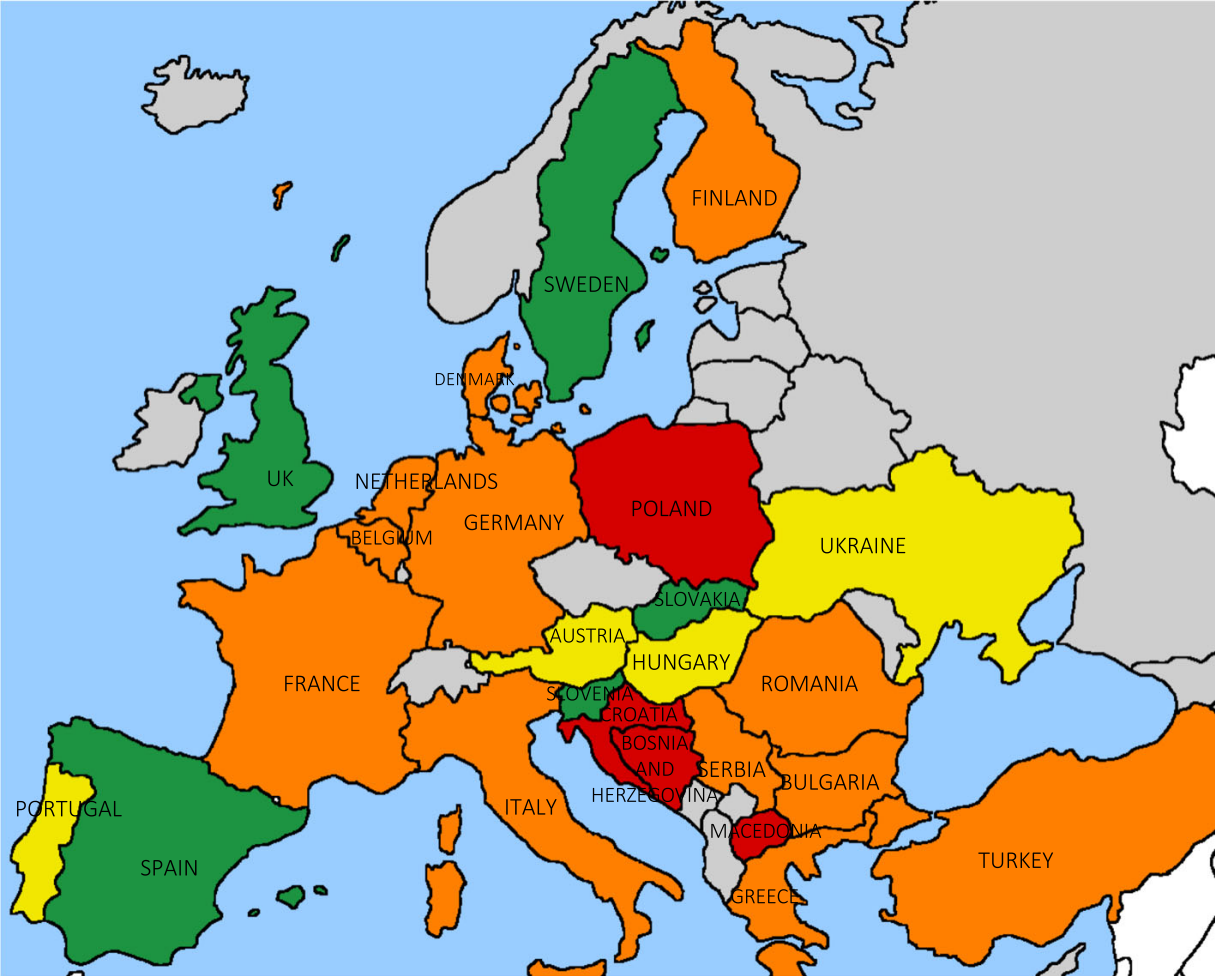
Availability and coverage of HCV treatment in European prisons. *Green*: HCV treatment available in all prisons; *yellow*: HCV treatment available in prisons, respondents provided data on the extent of availability; *orange*: HCV treatment available in prisons, extent unknown; *red*: no HCV treatment available; *gray*: not part of the Hep-CORE dataset

Twenty-one countries (84%) provide HCV treatment in prisons, while four countries (16%; Bosnia and Herzegovina, Croatia, Macedonia, Poland) do not. Only nine of the patient groups (36% of total) that reported the availability of HCV treatment in prisons could provide information on the proportion of prisons providing HCV treatment. Of these, five countries (20% of total; Slovakia, Slovenia, Spain, Sweden, UK) provide HCV treatment in all prisons, and the remaining four countries (16%; Austria, Hungary, Portugal, Ukraine) provide HCV treatment in less than half of the country’s prisons (Fig. 2).

### Needle and syringe programs

**Fig. 3:**
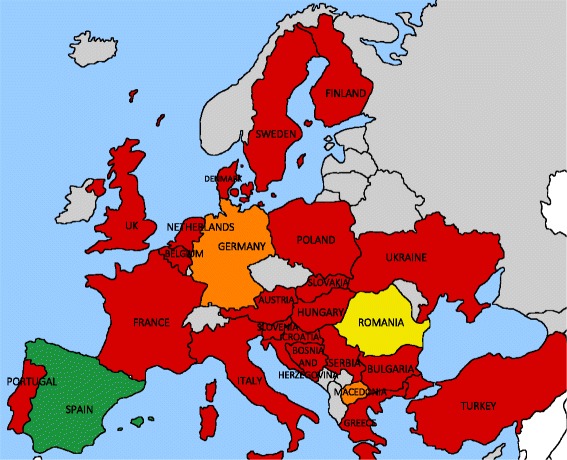
Availability and coverage of needle and syringe programs (NSPs) in European prisons. *Green*: NSP available in all prisons; *yellow*: NSP officially available in all prisons, but not in use due to enrollment requirements; *orange*: NSP available in at least one prison; *red*: NSP not available in prisons; *gray*: not part of the Hep-CORE dataset

Only two countries (8%; Spain, Romania) technically have NSPs available in prisons in all parts of the country. However, in Romania, due to the fact that prisoners need to file paperwork to register formally for the program, no prisoners are currently enrolled. Two countries (8%; Germany, Macedonia) have limited availability of NSPs in prisons. In Germany, this refers to availability in one prison out of approximately 180 total prisons, and in Macedonia, the availability is based on individual projects by nongovernmental organizations (NGOs) rather than programmatic activities of the state (Fig. 3).

Twenty-one countries (84%) do not have NSPs available in prisons, though the patient groups from Turkey, Sweden, and Denmark were unable to provide their opinion from a patient’s perspective for the Hep-CORE survey, explaining that they have limited access to information about prison health.

### Opioid substitution therapy programs

**Fig. 4:**
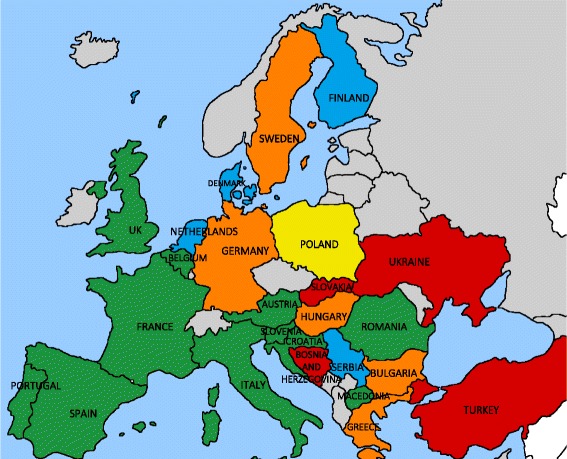
Availability and coverage of opioid substitution therapy (OST) programs in European prisons. *Green*: OST available in all prisons; *yellow*: OST officially available in all prisons, but low coverage due to additional requirement for abstinence; *blue*: OST available, but continuation only if treatment was started prior to incarceration; *orange*: OST available in some prisons; *red*: OST not available in prisons; *gray*: not part of the Hep-CORE dataset

Eleven countries (44%) have OST available in prisons in all parts of the country. In five countries (20%; Denmark, Finland, the Netherlands, Poland, Serbia), additional requirements for OST enrolment were reported by patient groups or the literature: in Poland, abstinence is a requirement, and therefore, coverage is low [34], and in Denmark, Finland, the Netherlands, and Serbia, OST is provided only if initiation began before incarceration [35]. In the UK, England and Wales have limitations on OST accessibility due to time-limited prescribing, whereas Scotland does not [18]. In five countries (20%; Bulgaria, Germany, Greece, Hungary, Sweden), OST is available in prisons in some parts of the country. Four countries (16%; Bosnia and Herzegovina, Slovakia, Turkey, Ukraine) do not have OST available in prisons in their country (Fig. 4).

### Levels of agreement between literature and patient group responses

There was some level of disagreement between the reviewed literature sources, as well as between literature sources and the patient group perspectives. The highest levels of uniform agreement between sources were in regard to treatment availability and availability of needle and syringe programs (21 countries (84%) and 20 countries (80%) with uniform agreement between sources, respectively). For the results from sources regarding testing/screening availability and coverage, as well as the provision of opioid substitution therapy, there was some level of disagreement between sources for 13 countries (52%) in both areas.

## Discussion

This study reviews the services available for harm reduction, testing/screening, and treatment of HCV infection in penitentiary settings. The latest available Hep-CORE data are sourced from ELPA member organizations that reported on barriers to access for high-risk populations in practice [31]. By comparing these data with the available literature, we were able to identify important discrepancies that were not apparent when reporting from a single source. The renewed attention to viral hepatitis in the wake of the advent of highly effective direct-acting antiviral (DAA) therapy, coupled with the adoption of the 2016 WHO *Global health sector strategy on viral hepatitis* [36] and the *Action plan for the health sector response to viral hepatitis in the WHO European Region* [37], has paved the way for action to reach the goal of eliminating viral hepatitis as a public health threat by 2030. Nonetheless, the stated attention and political commitments have rarely translated to action and implementation of services for one of the population groups with the highest prevalence of HCV infection: prisoners.

Counseling and testing for prisoners is a crucial entry point to eventual prevention, treatment, care, and support services for blood-borne viruses [38]. Tattooing in prisons with shared and unsterile needles and ink may also increase the risk of HCV acquisition. Programs to combat this would also contribute to harm reduction and disease prevention in the prison setting. In a project in penitentiary institutions in France and Luxembourg, for example, a health education project to promote safe tattooing has been piloted in order to train prisoners on safe tattooing and health considerations, regulate the tattoo trade, and provide safe instruments and medical oversight for prisoner tattoos. The project was proposed in one prison after researchers found that 50% of inmates surveyed had a tattoo, of which one out of three had been made during their prison stay [39].

On the other hand, results regarding prisoner access to testing and screening services are promising at first glance. In 21 of the 25 Hep-CORE study countries, testing for blood-borne viruses is officially possible in prison settings. However, in 16 countries (64%), there is no existing policy that guides the provision of testing/screening to prisoners as a high-risk population. Eight countries (32%) have such a policy, but the uptake of testing/screening is possibly suboptimal as the extent of the activity is unknown. In the UK, universal opt-out screening has been initiated as of 2015 in Scotland, 2016 in Wales, and April 2017 in England, and this will be expanded to all prisons in the coming years [40]. This is crucial, as a significantly higher uptake of testing has been shown to occur with opt-out procedures in prisons [41–43]. Furthermore, several trials have shown that improved screening with opt-out procedures and subsequent treatment with DAAs is cost-effective, despite the high costs of DAA treatments [44, 45].

In the past, studies have shown good outcomes of HCV treatment within penitentiary settings, both with pegylated-interferon-based regimens as well as with the new pan-genotypic once daily, all-oral 8-week DAA therapies [23, 44, 46–48]. The short duration of DAA treatments (from 8 to 12 weeks) has led to calls for increased HCV testing and treatment of prisoners [48]. However, the high cost of DAA therapy sets cost limitations on the expansion of HCV testing and treatment in prison as well as general population settings worldwide [45, 49].

As long as the health-care budget for prisoners is not regulated by the national or regional departments of health, this inequality of care will likely persist [18]. This is illustrated by the general lack of availability of DAA treatment in European prisons. Only five countries (Slovakia, Slovenia, Spain, Sweden, the UK) reported treatment to be technically available in all prisons. In this study, we did not examine the rates of HCV diagnosed versus treated prisoners. As availability is not equal to access, the actual situation is likely to be even more of a concern. Continued monitoring of these data in the future will be essential.

Provision of harm reduction, and thus prevention of further spread of blood-borne viruses, is still greatly lacking in penitentiary settings in Europe, and estimates of availability are poor [50, 51]. NSPs were found to be theoretically available in all prisons nationwide only in one country: Spain [34, 52, 53], a country that is also piloting efforts to reach full elimination of HCV in prison settings [54]. In most countries, NSPs were found to be entirely absent in prisons. This contrasts with access in the general population, outside the penitentiary setting, where NSPs are often available [18, 34]. The availability of OST in prisons was found to be more widespread.

Data on coverage of OST in prisons varied widely in the literature even within countries, depending on the year of publication of the study. This is likely due in part to the fact that availability, funding, and coverage of OST in prisons are highly unpredictable from year to year. Indeed, in some countries, services were shown to be available on paper, but limitations, stigmatization, and requirements for registration ensure that few, if any, prisoners are able to take advantage of the programs. In other countries, OST programs were available only in a limited number of prisons or regions (e.g., Germany, with some states nearly totally excluding OST as in Bavaria) [55]. This again is in contrast to the general community level where OST is more broadly accepted [18, 34]. This discrepancy in levels of care depending on the population in question is a clear violation of the human rights of prisoners [21].

Although we have the tools available to initiate the process of elimination of HCV, and incarceration settings are a clear opportunity to enroll patients in care, our study shows no improvements in care compared to the ACCESS study published in 2015 [35]. Moreover, there seems to be a lower coverage of OST programs and availability of HCV treatments. This is likely due to the fact that the data from the ACCESS study were obtained from official government sources, whereas data here factor in responses of patient organizations working on the ground. Nonetheless, we can clearly state that despite the documented high prevalence of HCV in prisons, internationally agreed-upon standards and recommendations for HCV testing/screening, care, and treatment, as well as prevention, have yet to be implemented [4, 24, 38]. In fact, no new NSPs in prison settings have been implemented in Europe in at least the past 10 years [38]. When we look in depth at service provision, OST programs continue to be slow and of poor quality, with limited uptake and heavy requirements for patients [38].

We report a number of limitations in the research that support this article. We contacted only one patient group in each country for the responses to the Hep-CORE survey. It is possible that the particular patient group was not able to accurately answer each question. However, those patient groups that reported lack of knowledge on a particular topic contributed to our understanding of which countries had restricted access to policy information, particularly in the prison health realm, and inaccurate government communication with concerned civil society organizations.

The findings from our study contribute considerably to the available knowledge on harm reduction in prisons as currently there are few studies that have addressed this gap in data. The Hep-CORE study is an on-going project intended to provide regular data to highlight the state of viral hepatitis policy in countries with participating patient groups, as well as change over time. The findings on viral hepatitis care in prison settings in Europe presented in this article serve as a baseline for future studies both in the Hep-CORE project and for other research efforts.

The results from the literature review for most of the 25 countries included in this study were based on only two large reports, which were published before and right after the development of recommendations for the management of HCV in prisons [35, 56]. Occasionally, there were discrepancies in the agreement between the different sources. We have highlighted countries where the level of disagreement was high and, as such, should be noted as places where access to clear policy information is difficult. The data available in the *Global state of harm reduction 2016* also reflect the paucity of up-to-date and reliable studies [18]. Publication of comprehensive results specifically on the outcomes of implemented harm reduction measures in prison settings is an unmet need and would further contribute to informing political and clinical decision-making.

With the goal of viral hepatitis elimination in mind, we urgently need to improve HCV care for prisoners [36]. Several programs have been developed that address specific barriers to care in prisons. The lack of specialists at prison sites has been shown to be successfully addressed using teleconferencing, videoconferencing, and email communication to connect specialists to primary care providers in prisons [57]. Nurse-led models have also effectively increased testing for treatment of and vaccination for blood-borne viruses [58, 59]. Treating prisoners in the pegylated-interferon era without restrictions based on expected incarceration time was also shown to be effective as long as, prior to release, prisoners received a timely referral to appropriate clinics for continuation of treatment [60]. Treatment using DAAs inherently overcomes this barrier due to shorter treatment times, which permits treatment to be completed fully during the incarceration period. Peer-based programs have also been shown to be effective in increasing HCV treatment awareness among prisoners [61].

As prisons provide a unique opportunity for HCV treatment, especially with the advent of DAA therapy, further efforts must be undertaken to pressurize governments into taking adequate care of their prisoners. Prisons, due to the prevalence of HCV within, as well as the number of prisoners who have a history of injecting drug use, are an ideal focal setting for micro-elimination efforts; however, targeted goals must be set in order to achieve this. Universal opt-out screening, in combination with high treatment uptake and the possibility of immediate linkage to care, is ultimately a cost-effective intervention [44]. Performing studies such as ours and publishing the data to inform governments from a multi-stakeholder perspective are crucial to stimulate the necessary policy change.

## Conclusion

Given the high prevalence of hepatitis C virus (HCV) among prisoners, disease prevention measures, such as opioid substitution therapy and needle and syringe programs, are currently insufficient in European prison settings. Only a minority of HCV-infected patients in prisons have access to direct-acting antiviral therapy, which can easily and effectively cure HCV. Scaled-up opt-out testing during or upon entry to prison settings, linked to prompt treatment, would be a major step towards the elimination of HCV and reduce the further spread of infection to people who inject drugs, other prisoners or to the general population upon release. Although recommendations have been formulated specifically in relation to HCV management in prisons, implementation efforts must be scaled up in order to eliminate HCV as a public health threat by 2030 in line with targets set by WHO and adopted by all European Member States.

## Abbreviations

DAA: Direct-acting antiviral
ELPA: European Liver Patients’ Association
HCV Ab+: HCV antibody positive
HCV: Hepatitis C virus
HIV: Human immunodeficiency virus
NSP: Needle and syringe program
OST: Opioid substitution therapy
PWID: People who inject drugs
REDCap: Research Electronic Data Capture
UK: United Kingdom
WHO: World Health Organization
